# The role of the transcription factor KLF16 in metabolic dysfunction associated fatty liver disease: regulatory linkages between lipid deposition and the expression of ATF4

**DOI:** 10.1080/07853890.2025.2566872

**Published:** 2025-10-01

**Authors:** Guanjun Cai, Xinyuan Cui, Wenyi Li, Shan Huang, Wenfang Peng

**Affiliations:** Department of Endocrinology, Tongren Hospital, Shanghai Jiao Tong University School of Medicine, Shanghai, China

**Keywords:** KLF16, MAFLD, ATF4, endoplasmic reticulum stress, lipid deposition

## Abstract

**Background and aims:**

The escalating prevalence of metabolic dysfunction-associated fatty liver disease (MAFLD) poses a significant global health burden. MAFLD is characterized by abnormal lipid accumulation in liver parenchymal cells and activation of endoplasmic reticulum stress. However, the effect of Krüppel-like factor 16(KLF16) on glycolipid metabolism has been limited. We investigated whether KLF16 could alleviate MAFLD through endoplasmic reticulum stress.

**Methods:**

HepG2 and mouse primary hepatocytes were treated with oleic acid (OA) to model MAFLD *in vitro*. siRNA was used to downregulate KLF16 expression in cells. C57/BL6J mice were fed a high-fat diet to model MAFLD *in vivo*, and AAV8 was used to regulate KLF16 and ATF4 expression. Western-blot, RT-qPCR, oil red O staining, and dual-luciferase reporter assays were used to explore the underlying mechanisms in MAFLD. T-test and ANOVA were used to compare the differences among groups.

**Results:**

KLF16 expression was upregulated in MAFLD models. KLF16 downregulation aggravates lipid deposition in liver cells. The expression of ATF4 protein was downregulated in MAFLD models with KLF16 knockdown. KLF16 transcriptionally regulates ATF4 expression through two binding sites. ATF4 overexpression alleviates lipid deposition exacerbated by KLF16 knockdown in mice.

**Conclusion:**

In the face of abnormal lipid deposition in MAFLD, liver cells can play a spontaneous protective role by upregulating KLF16, which can promote the expression of ATF4 at the transcriptional level, further affecting downstream lipid metabolism-related genes. KLF16 may serve as a promising target gene to improve the progression or prognosis of MAFLD.

## Introduction

With global economic and technological advancements, dietary habits and lifestyles have dramatically shifted. The rise in the consumption of high-fat and high-sugar foods, combined with sedentary behaviors and inadequate physical activity, has contributed to the escalating prevalence of metabolic dysfunction associated fatty liver disease (MAFLD). MAFLD is characterized by abnormal lipid accumulation in liver parenchymal cells and poses a significant health burden [1]. The liver, as a central metabolic organ, orchestrates the balance between fatty acid metabolism and circulation *via* several key processes, including de novo lipid synthesis, fatty acid β-oxidation, lipid transport, and lipid uptake [2]. ATP citrate lyase (ACLY), acetyl-CoA carboxylase (ACC1), fatty acid synthase (FASN), glycerol-3-phosphate acyltransferase (GPAT), acyl-CoA synthetase short-chain family member 2 (ACSS2), diacylglycerol O-acyltransferase (DGAT), stearoyl-CoA desaturase (SCD), and ELOVL fatty acid elongase 6 (ELOVL6) are the key genes in lipid synthesis and elongation. Acyl-CoA synthetase long-chain (ACSL), diacylglycerol cholinephosphotransferase (CPT), Medium-chain Acyl-CoA dehydrogenase (MCAD), Long-chain acyl-CoA dehydrogenase (LCAD), Adipose Triglyceride Lipase (ATGL), Monoglyceride Lipase (MAGL) plays an important role in lipid breakdown and fatty acid β-oxidation. Apolipoprotein (APO) is the main component of lipid transport carriers both inside and outside the liver. Fatty Acid Binding Proteins (FABP) and Fatty Acid Transport Proteins (FATP) are important genes that affect the uptake of exogenous lipids by hepatocytes. The complex lipid metabolism process is maintained in homeostasis through multi-gene regulation. However, excessive lipid accumulation surpassing the metabolic capacity of the liver can lead to chronic metabolic diseases such as MAFLD [3].

In China, the prevalence of chronic metabolic diseases has reached approximately 41.6%, among which more than 50% are suffering from MAFLD [4,5]. Unmanaged MAFLD can evolve into more severe conditions, such as steatohepatitis, liver fibrosis, and hepatocellular carcinoma [6]. Therefore, it is of great significance to maintain public health through further exploration of the mechanisms of MAFLD occurrence and development.

Existing research has shown that hepatic steatosis is the most significant pathological feature of MAFLD in its initial stages. Physiologically, trace lipid droplets in hepatocytes can store energy and protect cells from lipid toxicity; however, endoplasmic reticulum stress (ERS) and oxidative stress have been found to be activated in hepatocytes with excess lipid deposition. Endoplasmic reticulum stress is a physiological pathway that regulates endoplasmic reticulum homeostasis and plays a complex role in lipid accumulation in the liver [7]. Deletion of IRE1α-XBP1 in ERS promotes hepatic steatosis, whereas deletion of XBP1 has the same effect [8]. As for the PERK-EIF2α-ATF4 branch, basal phosphorylation of eIF2α prevents hepatic lipid deposition, and the deletion of ATF4 has a similar effect. The ATF6α branch had a protective effect on lipid deposition in the liver. In summary, ERS can be used to maintain cell stability and return cells to the normal state in response to abnormal lipid deposition. However, when ERS becomes chronic or acute ERS, it can exacerbate MAFLD and significantly accelerate disease progression [9]. Therefore, normal ERS plays an integral role in maintaining normal lipid metabolism in the liver.

In the study of chronic metabolic diseases, the Krüppel-like factor (KLF) family of transcription factors has been reported to be involved in many glucose and lipid metabolism processes. For example, KLF2 and KLF3 can affect lipid synthesis by participating in adipocyte differentiation, and KLF7 can participate in diabetes by impairing insulin synthesis and secretion in islet beta cells [10]. However, for Krüppel-like factor 16(KLF16), the main member of this family, current literature reports are limited to the role of KLF16 in the occurrence and development of glioblastoma, colorectal carcinomas, and other tumors [11]. Although KLF16 is known for its roles in glioblastoma and colorectal carcinomas, its specific function in glycolipid metabolism remains poorly understood, although it has been noted to mitigate fatty liver inflammation by targeting PPARα [12]. Therefore, this study aimed to explore the role of KLF16 in the regulation of lipid metabolism in MAFLD and its possible mechanisms.

## Materials and methods

### Animals

A total of 24 male C57BL/6J mice, aged 5 weeks, were obtained from Gem Pharmatech Co., Ltd. All animals were maintained under uniform standard conditions: a 12-h light/dark cycle, temperature maintained at 20–22 °C, access to clean drinking water, and a sufficient normal diet. Following a 1-week acclimatization period, mice were randomly divided into four groups (*n* = 6 each): i) normal diet (ND), ii) high-fat diet (HFD), iii) HFD+shKLF16, and iv) HFD+shKLF16 + ATF4. 6 mice in each group minimize animal use while allowing the final experimental data to be statistically analyzed.

Adeno-associated virus 8(AAV8) has strong tissue specificity to the liver. Once infected, it can stably exist for a long time. AAV8 does not integrate into the mouse genome, and has no association with any diseases that have been found. Therefore, we chose AAV8 as a vector and integrated the shRNA used for inhibiting KLF16 expression (AAV8-shRNA-KLF16) in mouse livers. Similarly, we constructed the corresponding AAV8 virus to overexpress the ATF4 gene in the livers of mice (AAV8-ATF4).

Groups iii) and iv) underwent AAV8-shRNA-KLF16 administration *via* tail vein injection after isoflurane anesthesia (2E + 11VG per mouse), whereas groups i) and ii) received AAV8-control in a similar manner. After the injection, all mice were returned to their respective cages. For the subsequent 12 weeks, groups ii), iii), and iv) were fed a high-fat diet (D12492, sourced from Research Diets Inc., composed of 60% fat, 20% carbohydrate, and 20% protein), which is known to induce steatosis, but not severe liver injury (characterized by inflammation and fibrosis). At the 12th week, group iv) mice received AAV8-ATF4, and group iii) mice received AAV8-pcDNA3.1, following the previously described isoflurane anesthesia and tail-vein injection protocol.

**Figure 1.**
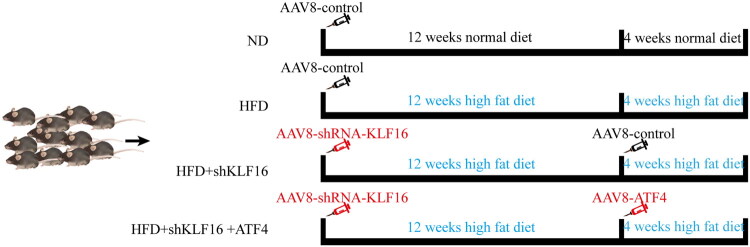
Mice experiment process plan diagram.

After an additional 4-week feeding period, all mice were euthanized by cervical dislocation after a 6-hour fasting period. The experimental procedure is illustrated in Figure 1. Blood samples were collected from the retro-orbital plexus after eyeball removal, and serum samples were subsequently obtained by centrifuging the blood at 3000 rpm for 15 min after a 1-hour resting period and collecting the supernatant. After the mouse limbs were fixed, liver samples were obtained by abdominal surgery, and paraffin sections and frozen sections were prepared.

### Hematoxylin and eosin (H&E) staining and oil red O staining

For HE staining, the procedure began by allowing the frozen sections to acclimate to room temperature for 15 min. They were then fixed in a 95% ethanol solution for 1 min. Subsequently, the slides were immersed in a hematoxylin dye bath for 5 min. After hematoxylin staining, the excess dye was washed away under running water. The sections were briefly immersed (for 2 s) in a 1% hydrochloric acid-ethanol solution, followed by a water rinse. The slides were then placed in an eosin dye bath for 1-minute staining. After eosin staining, the excess dye was removed by washing the sections with water. The sections were dehydrated using a series of gradient alcohol concentrations and cleared using xylene. Finally, the slides were sealed with neutral gum to maintain the staining.

Frozen slices were taken from a −80 °C refrigerator and left for 15 min at room temperature. The sections were then immersed in 4% paraformaldehyde for 30 min. The sections were rinsed with distilled water before immersion in a 60% isopropyl alcohol solution for 3–5 min. The sections were then directly transferred into an Oil Red O working solution for staining, which lasted for 10 min. After staining, the sections were re-immersed in 60% isopropyl alcohol solution for 3-minute immersion, followed by washing with distilled water. Finally, the stained sections were sealed to maintain the staining results.

### Biochemical indicators analysis

The levels of total cholesterol (TC), triglyceride (TG), high-density lipoprotein (HDL), low-density lipoprotein (LDL), ALT, and AST in the mouse plasma were assessed using an Automatic Biochemical Analyzer (Chemray 800, Rayto Life and Analytical Sciences Co., Ltd.). The intrahepatic contents of TG and TC were quantified using kits supplied by the Nanjing Jiancheng Bioengineering Institute (A111-1-1, A110-1-1), with the procedures carried out in strict accordance with the manufacturer’s instructions.

### Cell line and primary hepatocytes culture

Human HepG2 cell line (from American Type Culture Collection, ATCC) and mouse primary hepatocytes were used in this study. They have been used to model MAFLD *in vitro* to explore the role of KLF16 in lipid regulation in liver parenchymal cells. The HepG2 cells were maintained in DMEM (Gibco, 11965084) supplemented with 10% fetal bovine serum (Gibco, 10270-106) and 1% penicillin-streptomycin (Gibco, 15140122) at 37 °C in a humidified atmosphere containing 5% CO2. The medium was changed every other day. The HepG2 cell was sub-cultured every 4–5 days when the confluence was about 80–95%, at a ratio of 1:3 or 1:2. The primary cells were isolated from the livers of 8-week-old C57/BL6J mice according to methods outlined in previously published literature [13,14]. The cells were maintained in DMEM (Gibco, 11965084) supplemented with 10% fetal bovine serum (Gibco, 10270-106) and 2% penicillin-streptomycin (Gibco, 15140122) in the same culture environment as HepG2.

The first step in modeling MAFLD *in vitro* is to replace the medium with a serum-free medium. Both the Human HepG2 cell line and mouse primary hepatocytes were treated with 400 µM oleic acid (OA, MB2952-1; Merck Millipore) for 24 h to induce lipid accumulation, simulating the MAFLD condition. After treatment, cells were subjected to western blot analysis or real-time quantitative polymerase chain reaction (qPCR) to investigate the effects of OA on KLF16 expression and lipid accumulation.

Human embryonic kidney (HEK)-293T cells (from American Type Culture Collection, ATCC) were used as cell vectors in the dual-lucifease reporter due to their high amplification efficiency and high transfection efficiency. They were maintained in RPMI 1640 (Gibco, C11875500BT) supplemented with 10% fetal bovine serum (Gibco, 10270-106) and 1% penicillin-streptomycin (Gibco, 15140122) at 37 °C in a humidified atmosphere containing 5% CO2. The medium was changed every other day.

### Cell transfection

The cells were seeded in a 6-well plate at an appropriate density. After 24 h of incubation, the medium was replaced with Opti-MEM to prepare cells for transfection. Subsequently, siRNA or plasmids were introduced into the target cells using the Lipofectamine 3000 transfection kit (Invitrogen, L3000-015). The transfection time was 8–12 h, and the transfected cells were then replaced with normal cell medium.

### RT-qPCR

TRIzol RNA extraction reagent was used to isolate total RNA from liver tissue or liver cells. Following RNA extraction, a high-efficiency reverse transcription kit (TOYOBO, FSQ-301) was used to synthesize cDNA, which was essential for RT-qPCR analysis. The expression levels of specific genes across various samples were quantified using RT-qPCR, facilitated by the ChamQ Universal SYBR qPCR Master Mix (Vazyme, Q711-02) and multiple primers. Detailed information regarding the primer sequences is provided in Table S1(A–B).

### Western blot

RIPA lysis buffer (Beyotime Biotechnology, P0013C) supplemented with 1% protease inhibitor PMSF (Beyotime Biotechnology, ST505) was used for the extraction of total proteins from liver tissue or liver cells. Protein concentrations were quantified using a BCA protein assay kit (KeyGEN Biotech, KGP903). Proteins were resolved by SDS-PAGE (Beyotime Biotechnology, P0012A) and transferred onto polyvinylidene fluoride membranes (Servicebio Technology, WGPVDF22). Ladders used in the western blot were from Epizyme Biotech (WJ102). The PVDF membrane was immersed in 5% skim milk powder solution at room temperature for 1 h for blocking. The membrane was then removed and rinsed with TBST solution three times for 5 min each. The membranes were then incubated overnight at 4 °C with specific primary antibodies. After another series of washes, the membranes were incubated with HRP-conjugated secondary antibodies at room temperature for 1 h. After washing, the membrane was developed using an Extremely Sensitive ECL Chemiluminescence Kit (NCM Biotech, P10060), and immunoreactive bands were detected using an automatic chemiluminescence image analysis system. For quantification of the bands, ImageJ software (Bethesda) was used. Information regarding the antibodies is provided in Table S1C.

### Dual-luciferase reporter

The gene sequence of KLF16 was cloned and subsequently inserted into the pcDNA3.1. The eukaryotic vector expressing KLF16 was termed pcDNA3.1-KLF16. To examine the affinity of the transcription factor KLF16 for the ATF4 promoter sequences, recombinant plasmid PGL3, which contains the predicted binding sites or mutated ATF4 promoter sequences, was constructed and transfected into (HEK)-293T with pcDNA3.1-KLF16. The recombinant plasmid constructed was termed PGL3-ATF4, PGL3-MUT1, PGL3-MUT2, and PGL3-MUT1 + 2. The expression level of luciferase in cells increased with the growth of the affinity of the transcription factor KLF16 for the promoter sequences from different groups, ultimately affecting the relative fluorescence intensity of the cell homogenates of different groups. (HEK)-293T cells were cultured in a 6-well plate as a cell carrier. PcDNA3.1 and PGL-3 plasmids were co-transfected according to the guidelines provided by the Dual-Luciferase Reporter Assay Kit (KeyGEN BioTECH, KGAF040).

### Statistical analysis

Statistical analyses were performed using GraphPad Prism 8 software. The Shapiro–Wilk test was used to assess data normality. Homogeneity of variance was evaluated using the Brown–Forsythe test. For comparisons between two groups, an unpaired two-tailed Student’s *t*-test was applied. For comparisons among more than two groups, one-way analysis of variance (ANOVA) followed by Tukey’s post hoc test was performed. A *p*-value <0.05 was considered statistically significant. All experiments were repeated at least three times, and data are presented as mean ± standard deviation (SD).

## Results

### The expression of KLF16 is up-regulated in MAFLD models

**Figure 2.**
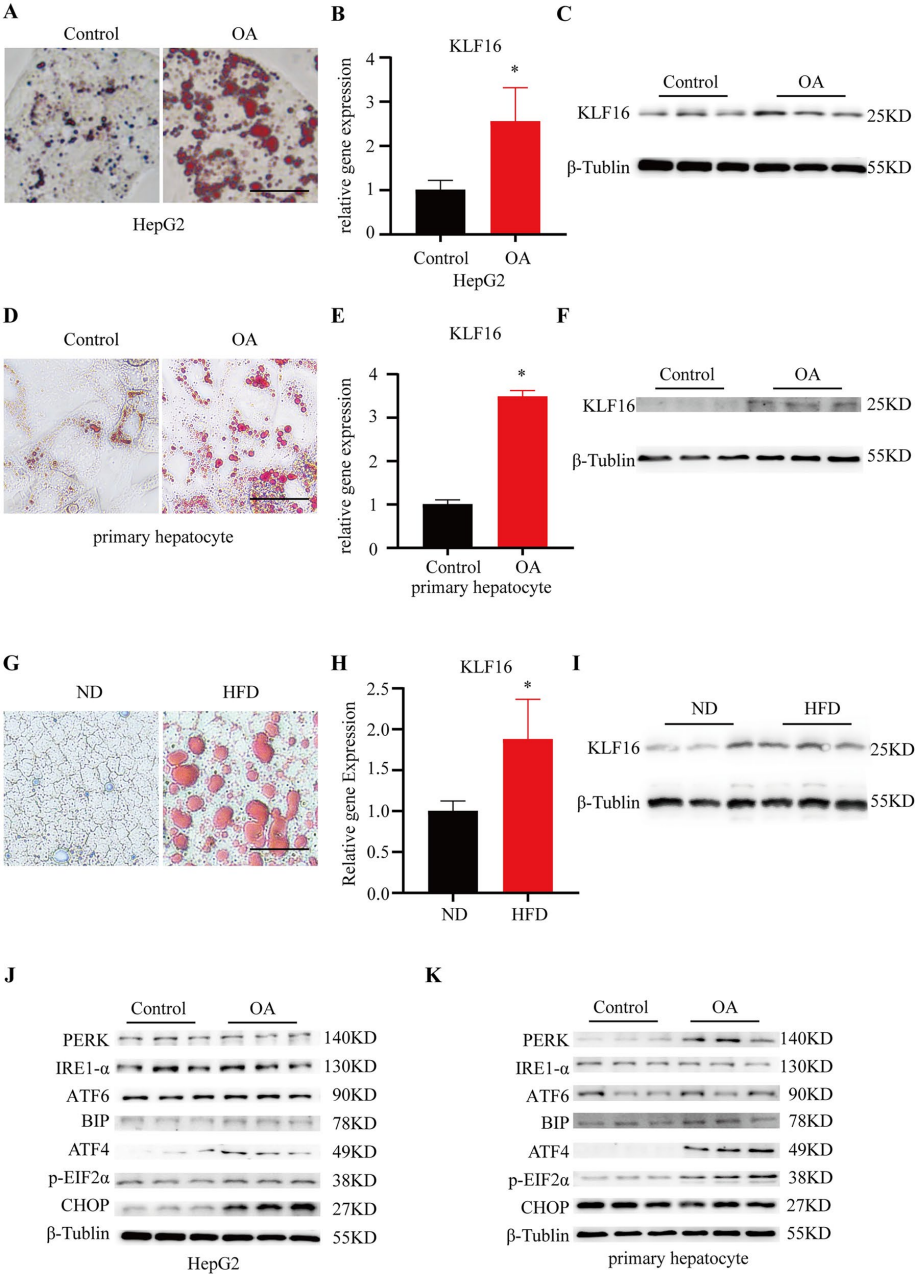
The expression of KLF16 is up-regulated in MAFLD models (A) oil red O staining of HepG2 cell. Bar, 200um. (B) Expression of gene KLF16 in HepG2 (*n* = 3). (C) Expression of protein KLF16 in HepG2 (*n* = 3). (D) oil red O staining of primary hepatocyte. Bar, 200um. (E) Expression of gene KLF16 in primary hepatocyte (*n* = 3). (F) Expression of protein KLF16 in primary hepatocyte (*n* = 3). (G) oil red O staining of mice liver sections. Bar, 200um (*n* = 3). (H) Expression of gene KLF16 in mice livers (*n* = 3). (I) Expression of protein KLF16 in mice livers (*n* = 3). (J–K) Western blot is done to check proteins involved in ER stress in HepG2 and primary hepatocyte from control or OA induced subjects (*n* = 3). Statistical analysis: for panels A-K, data were analyzed using an unpaired two-tailed student’s t-test to assess statistical significance. Data are expressed as mean ± SD. *p < 0.05, **p < 0.01.

Following established protocols, we treated HepG2 cells and primary hepatocytes with oleic acid (OA) and subjected C57/BL6J mice to a 12-week high-fat diet regimen. Oil Red O staining revealed a marked increase in lipid accumulation in OA-treated liver cells, with the quantity and variety of lipid droplets significantly surpassing those in the control group, as depicted in Figure 2 ADG. This lipid accumulation closely resembled the hepatic steatosis observed in MAFLD. Post-lipid accumulation, a significant upregulation of KLF16 was detected in both the HepG2 cell line and mouse primary hepatocytes compared to their untreated counterparts (*p* < 0.05) (Fi2.BCEF). A similar increase in KLF16 expression was noted in HFD-fed mice relative to the controls (Figure 2H,I).

Moreover, hepatocytes with lipid deposition exhibit activation of endoplasmic reticulum (ER) stress. Consistent with the literature, upon MAFLD insult, the liver expression of ER stress-related proteins was altered, notably ATF4 and p-EIF2α, which surged in both cellular and mouse MAFLD models (Figure 2J,K), along with varying changes in other associated proteins (Figure 1A,B). Given the relatively unexplored role of KLF16 in glycolipid metabolism, these findings suggest that KLF16 is involved in lipid metabolism in MAFLD. Recognizing that ER stress activation is a prevalent response to aberrant lipid metabolism in hepatocytes, we hypothesized that KLF16 regulates lipid homeostasis by directly modulating the expression of ER stress-related proteins.

### Knockdown of KLF16 induces increased hepatic lipid deposition

**Figure 3.**
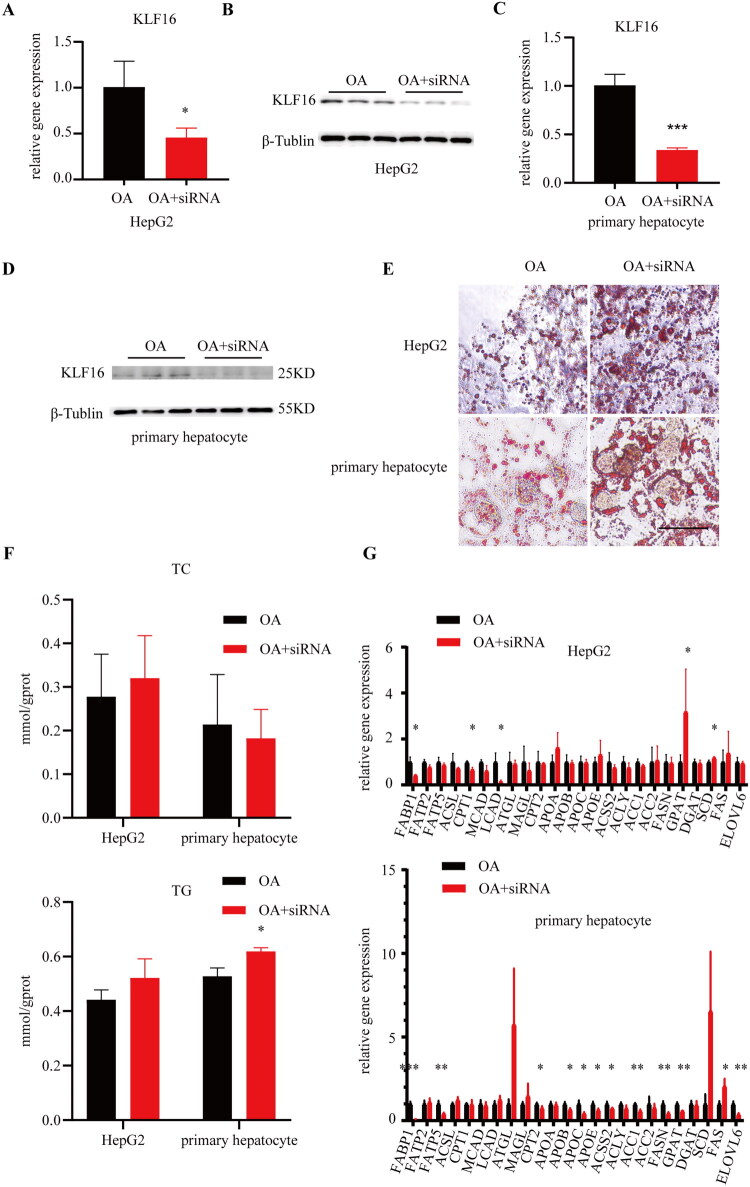
Knockdown of KLF16 induces increased hepatic lipid deposition (A) RT-qPCR of HepG2 treated with siRNA-KLF16 to detect the gene expression of KLF16(*n* = 3). (B) Western blot of HepG2 cells treated with siRNA-KLF16 to detect the protein expression of KLF16(*n* = 3). (C) RT-qPCR of primary hepatocytes treated with siRNA-KLF16 to detect the gene expression of KLF16(*n* = 3). (D) Western blot of primary hepatocytes treated with siRNA-KLF16 to detect the protein expression of KLF16(*n* = 3). (E) Oil red O staining of OA-treated HepG2 cells and primary hepatocytes, with or without KLF16 knockdown. (F) Intracellular TC and TG levels were measured in HepG2 cells and primary hepatocytes with and without KLF16 knockdown (*n* = 3). (G) RT-qPCR was performed to identify genes involved in lipid metabolism in HepG2 cells and primary hepatocytes from OA or OA+siRNA-KLF16 induced subjects (*n* = 3). Statistical analysis: for panels A–G, data were analyzed using an unpaired two-tailed student’s t-test to assess statistical significance. Data are expressed as mean ± SD. *p < 0.05, **p < 0.01, ***p < 0.001.

To assess the impact of altered KLF16 expression on lipid metabolism homeostasis in the liver, siRNA targeting KLF16 was designed, with sequences for both mouse and human siRNA-KLF16, as detailed in Table 1. The knockdown efficacy of siRNA was confirmed in both cell types *via* RT-qPCR and western blot analyses (Figure 3A–D). HepG2 cells with KLF16-specific knockdown showed more significant and severe intracellular lipid deposition under the same OA induction conditions. A similar pattern was observed in primary mouse liver cells (Figure 3E).

**Table 1.** siRNA-KLF16 sequence.

Gene	Species	Sequence (5′-3′)
KLF16	human	GCGCUAGUGAGAUGCCUUAGUTT ACUAAGGCAUCUCACUAGCGCTT
KLF16	mouse	CATGGCTGCGCCAAAGCCTATTACATT UGUAAUAGGCUUUGGCGCAGCCAUGTT

When evaluating the primary lipid components, cholesterol and triglycerides (TG), KLF16 knockdown did not significantly alter intracellular cholesterol levels in HepG2 or primary mouse liver cells (*p* > 0.05). However, downregulation of KLF16 expression in hepatocytes can increase TG content. Specifically, in HepG2 cells, intracellular TG concentrations increased following KLF16 downregulation (*p* > 0.05), whereas in mouse primary liver cells, a significant increase in TG was noted after post-KLF16 knockdown (*p* < 0.05) (Figure 3F).

Furthermore, analysis of lipid metabolism-related genes in HepG2 cells revealed that KLF16 knockdown suppressed the expression of genes associated with fatty acid β-oxidation, Carnitine Palmitoyltransferase I (CPT1), and Acyl-CoA Dehydrogenase Long Chain (LCAD). Upregulated Glycerol-3-Phosphate Acyltransferase (GPAT), a gene linked to TG synthesis, was correlated with the observed increase in hepatocyte TG levels (Figure 3G). Conversely, fatty acid-binding protein 1 (FABP1), a gene involved in liver lipid absorption, was downregulated, whereas Stearoyl-CoA Desaturase (SCD), a gene responsible for desaturating fatty acids, was upregulated. A similar trend was observed in mouse primary hepatocytes, with genes involved in fatty acid absorption and transport (FABP1) and fatty acid transport protein 5 (FATP5). Genes involved in fatty acid synthesis—Acetyl-Coenzyme A Synthetase2 (ACSS2), Fatty Acid Synthase (FASN), GPAT, Fatty Acyl-CoA Elongase (ELOVL6)–were downregulated, indicating a suppression of lipid accumulation. However, genes implicated in fatty acid β-oxidation—Carnitine Palmitoyltransferase II (CPT2), Acetyl-CoA Carboxylase Beta (ACC2), lipid export from the liver, Apolipoprotein B (APOB), Apolipoprotein C (APOC), Apolipoprotein E (APOE), and mitochondrial function-apoptosis signaling receptor (FAS) were also downregulated, highlighting a disruption in lipid metabolism and transport within the liver. Collectively, these changes in lipid metabolism-related genes suggest abnormal lipid deposition in hepatocytes under KLF16 downregulation.

### Transcriptional factor KLF16 regulate expression of ATF4

**Figure 4.**
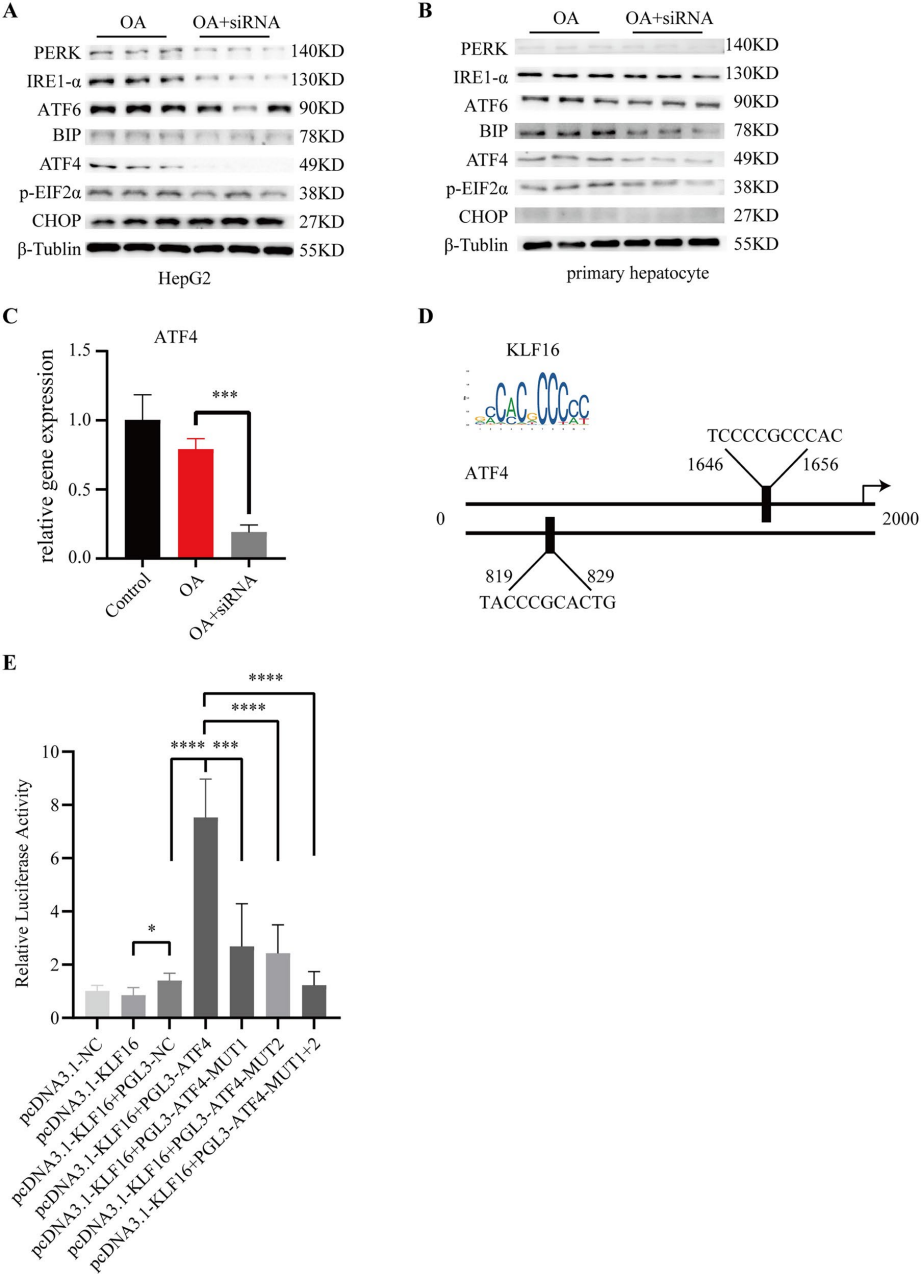
Transcriptional factor KLF16 regulate expression of ATF4 (A–B) Western blot is done to check proteins involved in ER stress in HepG2 and primary hepatocyte from OA or OA+siRNA-KLF16 induced subjects (*n* = 3). (C) Expression of gene ATF4 in primary hepatocyte from OA or OA+siRNA-KLF16 induced subjects (*n* = 3). (D) The sequence of ATF4 promoter sites predicted by JASPAR database that KLF16 may bind. (E) Dual-luciferase reporter experiment to test binding activity of KLF16 with ATF4 promoter sequence or mutated ATF4 promoter sequence (*n* = 3). Statistical analysis: Panels A-C were analyzed using an unpaired two-tailed student’s *t*-test, whereas panel E was analyzed using one-way ANOVA followed by tukey’s post hoc test. Data are expressed as mean ± SD. *p < 0.05, **p < 0.01, ***p < 0.001, ****p < 0.0001.

In a cellular MAFLD model, increased lipid deposition in the cells was observed in association with low KLF16 expression. This was accompanied by altered expression patterns of ER stress-related proteins (Figure 4A,B, Figure S2A,B). Specifically, in HepG2 cells, the expression of PERK, IRE1-α, and ATF4 was downregulated when ER stress was activated, and KLF16 expression was artificially reduced. Similarly, IRE1α, BIP, and ATF4 proteins were downregulated in primary mouse hepatocytes. At the protein level, both cell types exhibited downregulation of IRE1-α and ATF4, in conjunction with decreased KLF16 expression. RT-qPCR experiments showed that downregulation of KLF16 expression was accompanied by downregulation of ATF4 expression at the RNA level (Figure 4C). These results suggest that KLF16 may influence ATF4 transcriptional expression.

To confirm whether KLF16 is directly involved in transcriptional regulation of ATF4, we used the JASPAR (https://jaspar.elixir.no/), which is an open-access database containing manually curated high-quality and non-redundant DNA-binding profiles for transcription factors (TFs) among different species. According to the results of the analysis, two sequences from the ATF4 promoter sequence were predicted to have good binding ability with KLF16(Fig4D). The promoter sequence containing or mutating these two predicted binding sites was integrated into the PCL-3 plasmid. (MUT1= Mutations in sequence GTCACGCCCAT, MUT2= Mutations in sequence TCCCCGCCCAC, MUT1 + 2= Mutations in both sequences). In 293 T cells, co-transfection of pcDNA3.1-KLF16 and the PGL-3 plasmid with the ATF4 promoter significantly enhanced cellular luciferase activity, indicating that KLF16 can bind to the ATF4 promoter and upregulate ATF4 (Figure 4E).

However, through mutating the ATF4 promoter sequences (MUT1 and MUT2) to lose its affinity for KLF16, a marked reduction in luciferase activity was observed, indicating that both predicted sites could be recognized and bound by KLF16. When both sites were mutated, luciferase activity decreased further and aligned with the activity observed following transfection with the PGL3 plasmid alone. This demonstrates that these two binding sites are pivotal for KLF16’s transcriptional regulation of ATF4.

### Overexpression of ATF4 alleviates hepatic lipid deposition caused by down-regulation of KLF16

**Figure 5.**
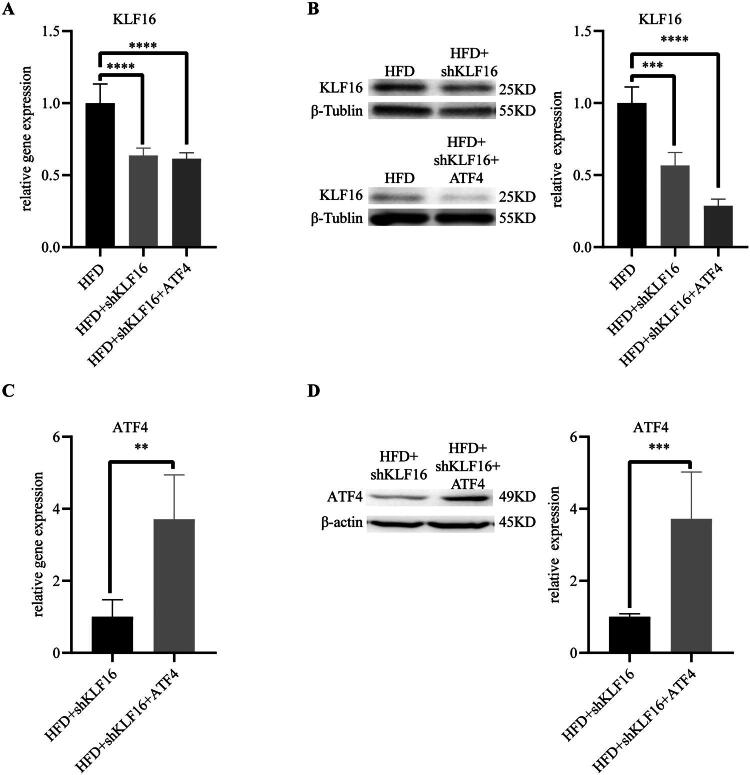
Overexpression of ATF4 alleviates hepatic lipid deposition caused by down-regulation of KLF16 (A) RT-qPCR to test KLF16 expression in mice infected with or without AAV8-shRNA-KLF16 (*n* = 6). (B) KLF16 protein expression in mice infected with AAV8-shRNA-KLF16 (*n* = 6). (C) ATF4 expression in mice infected with AAV8-ATF4 (*n* = 6). (D) ATF4 protein expression in mice infected with AAV8-ATF4 (*n* = 6). Statistical analysis: Panels A–B were analyzed using one-way ANOVA followed by tukey’s post hoc test, and panels C–D were analyzed using an unpaired two-tailed student’s *t*-test. Data are expressed as mean ± SD. *p < 0.05, **p < 0.01, ***p < 0.001, ****p < 0.0001.

KLF16 expression levels were verified by western blotting (WB) and RT-qPCR. As shown in Figure 5A,B, mice receiving tail vein injections of AAV8-shKLF16 showed significantly downregulated KLF16 expression in the liver compared to those on a high-fat diet alone. Concurrently, mice injected with AAV8-ATF4 showed markedly elevated ATF4 levels in their livers (Figure 5C,D), suggesting a potential compensatory mechanism for the effects of KLF16 knockdown.

**Figure 6.**
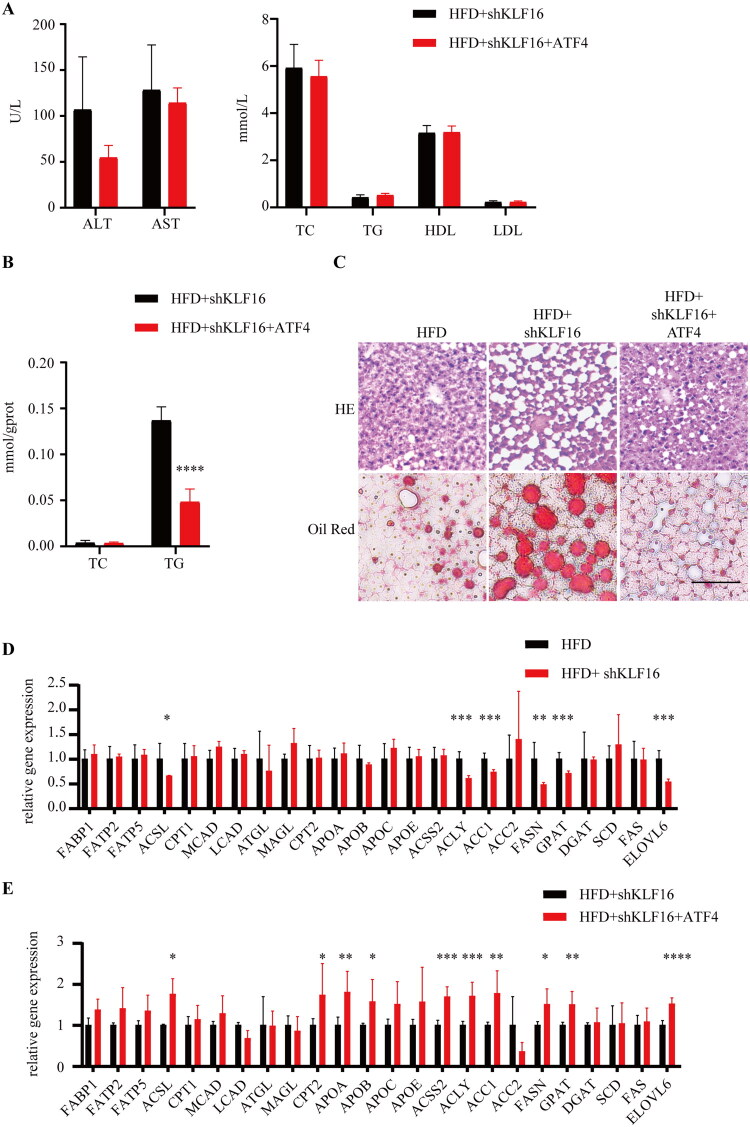
Overexpression of ATF4 alleviates hepatic lipid deposition caused by down-regulation of KLF16 (A) serum ALT, AST, TC, TG, HDL, LDL in mice (*n* = 6). (B) Liver TC and TG levels in the mice (*n* = 6). (C) Oil red O staining of mouse liver sections. (D–E) RT-qPCR of lipid metabolism-related genes in mouse livers from the respective groups (*n* = 6). Statistical analysis: Panels A–B and D–E were analyzed using an unpaired two-tailed student’s *t*-test. Data are expressed as mean ± SD. *p < 0.05, **p < 0.01, ***p < 0.001, ****p < 0.0001.

Subsequent analysis involved measuring serum levels of ALT, AST, TC, TG, HDL, and LDL in mice, with no significant differences observed (Figure 6A). In the livers of mice, no difference in TC content was observed between the HFD + shKLF16 and HFD + shKLF16 + ATF4 groups (Figure 6B). However, with ATF4 overexpression in the HFD + shKLF16 + ATF4 group, the TG content in the liver of mice showed a decreasing trend. HE staining, KLF16 knock-down reveals an increase in the number and size of cavities in the livers of mice in the HFD + shKLF16 group compared to the HFD group. ATF4 overexpression resulted in fewer and smaller vacuoles (Figure 6(C)). Oil Red O staining corroborates this, showing a decrease in both the size and quantity of lipid droplets post-ATF4 overexpression (Figure 6(C)).

RT-qPCR analysis revealed that genes related to lipid synthesis, ATP Citrate Synthase (ACLY), Acetyl-CoA Carboxylase Alpha (ACC1), FASN, GPAT, and ELOVL6, were downregulated in the HFD + shKLF16 group, indicating suppressed liver lipid production. However, Long-Chain Acyl-CoA Synthetase (ACSL), a crucial enzyme involved in fatty acid metabolism, was also downregulated, suggesting impaired lipid metabolism pathways. In the HFD + shKLF16 + ATF4 group, upregulation of ACSL, CPT2, APOA, and APOB resulted in enhanced liver fatty acid β-oxidation and lipid transport compared with the HFD + shKLF16 group. The upregulation of ACSS2, ACLY, ACC1, FASN, GPAT, and ELOVL6 suggests an increase in de novo lipid synthesis in the liver. The collective effect in the HFD + shKLF16 + ATF4 group was a reduction in liver lipid deposition (Figure 6(D,E)).

## Discussion

The rapidly increasing incidence and prevalence of MAFLD raise serious health concerns as it is intricately linked to various chronic metabolic diseases. Poorly managed MAFLD can even increase the potential risk of liver cancer [15]. Despite extensive research, its pathogenesis remains unclear, and effective pharmacological interventions are yet to be established [16]. In this study, we aimed to explore the role of KLF16 in MAFLD.

First, KLF16 was found to exhibit an upregulated expression trend in both *in vitro* and *in vivo* models of MAFLD. During the construction of the MAFLD model, lipid deposition in both HepG2 and mouse primary hepatocytes was further observed to activate the PERK-EIF2α-ATF4 pathway, manifested as upregulation of PERK, ATF4, and p-EIF2α expression. ATF4 improved the adaptability of cells to lipids by activating downstream REDOX reactions [7]. In addition to that, in HepG2 cells, the expressions of BIP and CHOP were also upregulated. It was hypothesized that the hepatoma cell line HepG2 was more sensitive to lipid deposition, which intensified endoplasmic reticulum stress, further promoting cell apoptosis [17].

To further explore the role of KLF16 in MAFLD, we artificially knocked down KLF16 expression in HepG2 cells and mouse primary hepatocytes. It was found that after the same concentration of oleic acid induction for the same time, intracellular lipid deposition was more severe in the group with low KLF16 expression. In HepG2, KLF16 knockdown is accompanied by a decline of FABP1, showing a weakening of lipid intake. The down-regulation of CPT1, LCAD limits the β-oxidation process of fatty acids within cells. The upregulation of GPAT and SCD indicates the promotion of lipid synthesis processes. In HepG2 cells, the knockdown of KLF16 weakens the uptake and consumption of lipids by hepatocytes, promoting the synthesis of lipids instead. In mouse primary hepatocytes, genes related to lipid metabolism, including de novo lipid synthesis (ACSS2, ACC1, FASN, GPAT, ELOVL6), fatty acid β-oxidation (CPT2), lipid transport (apolipoprotein), and lipid uptake (FABP1, FATP5), all decreased along with the inhibition of KLF16 expression. This result suggests that the inhibition of KLF16 expression caused lipid metabolism in the *in vitro* mouse MAFLD cell model to be in a stagnant state, and the lipid circulation homeostasis is disrupted.

There is no significant difference in TC content in lipid deposition, but TG content increased with the knockdown of KLF16. At the same time, along with the decreased expression of KLF16, ER stress-related proteins activated by lipid deposition in cells were also affected. In HepG2 cells, the expressions of PERK, ATF4, and IRE1-α were downregulated. In primary mouse hepatocytes, the expressions of IRE1-α, BIP, and ATF4 were downregulated. All indicate impaired activation of the endoplasmic reticulum stress. Among them, IRE1-α is mainly responsible for degrading mRNA, thereby alleviating the protein load of the endoplasmic reticulum. IRE1-α downregulation weakened the self-regulatory ability of the endoplasmic reticulum, which may be one of the reasons for the intensification of intracellular lipid deposition. Combined with the above experimental results, ATF4 was found to have the same expression trend as KLF16.

Previous studies have suggested that endoplasmic reticulum (ER) stress activation is a physiological response to abnormal lipid droplet deposition in liver cells [18]. ER stress, essential for the correct folding and assembly of polypeptide chains, is activated when misfolded proteins accumulate to restore ER function [19]. This process is mediated by interactions between key ER stress response proteins [20]. For example, in the PERK-EIF2α-ATF4 pathway, the downstream ATF4 protein is synthesized in response to ER stress, initiating gene transcription to mitigate ER abnormalities. ATF4 can also reverse the dephosphorylation of upstream EIF2α, thereby alleviating ER stress [21]. However, excessive ATF4 expression or unresolved ER stress can trigger cell death by promoting CHOP expression [22,23].

Further experiments revealed that KLF16 transcriptionally regulates the expression of ATF4. The results of the dual luciferase reporter assay showed that the transcription factor KLF16 promotes the expression of ATF4 mainly by binding the GTCACGCCCAT and TCCCCGCCCAC site sequences of the ATF4 gene promoter. The knockdown of KLF16 inhibits the expression of ATF4. As an important molecule in the unfolded protein reaction (UPR) process, ATF4 decline reduces the tolerance of hepatocytes to lipid stimulation, intensifies the activation of endoplasmic reticulum stress, and promotes cell death, thereby aggravating lipid deposition in the liver.

In order to study how KLF16 regulates ATF4 and thereby affects the lipid metabolism within hepatocytes, *in vivo* animal experiments of MAFLD were designed. ATF4, an endoplasmic reticulum stress-related protein, also plays a role in regulating hepatic lipid metabolism [24]. Based on previous studies, Mice completely knocked out for ATF4 showed resistance to age-induced and high-fat diet-induced obesity, accompanied by reduced production of apolipoprotein and VLDL in the liver, resulting in an obstruction of the outward transport of lipids in the liver [25–26]. By constructing *in vivo* models of MAFLD, artificially lowering KLF16 expression, and overexpressing ATF4, significant and widespread changes in lipid metabolization-related genes in the mouse liver were observed, which is consistent with existing literature reports [27,28].

In the physiological state, fatty acids in hepatic parenchymal cells combine to form triglycerides and cholesterol esters, which are stored in lipid droplets at physiological levels that are dynamically turned over within cells through continuous cycles of synthesis, growth, and degradation, thereby providing energy and serving as precursors for cellular components [29,30]. In the MAFLD animal model, however, the inhibition of KLF16 expression results in a significant increase in TG levels within hepatic parenchymal cells, suggesting excessive lipid deposition. Meanwhile, ACSL, ACLY, ACC1, FASN, GPAT, and ELOVL6 gene expression were observed to decline along with the downregulation of KLF16. It suggests that lipid synthesis in the liver is blocked. As an important organ for lipid metabolism, the knockdown of KLF16 inhibits the liver from converting a variety of fatty acids into neutral lipids, which have lower toxicity. Therefore, a large amount of lipids stay in the liver cells and cannot be metabolized, thereby damaging the liver parenchymal cells and disrupting the normal structure of the liver.

Along with the overexpression of ATF4 in the HFD+shKLF16 + ATF4 group, it was found that lipid deposition in the liver improved significantly. Also, the expression of genes related to lipid transport (APOA, APOB), genes related to lipid synthesis (ACSS2, ACLY, ACC1, FASN, GPAT, ELOVL6), Genes related to lipid oxidation (ACSL, CPT2), were all upregulated. Based on the above results, it was hypothesized that KLF16 promotes the expression of ATF4 at the transcriptional level in MAFLD, thereby enhancing the tolerance of hepatic parenchymal cells to lipid deposition and improving lipid deposition in the liver through the dynamic balance of lipid circulation within hepatic parenchymal cells. Restoration of the regulatory ability of the endoplasmic reticulum transforms different fatty acids into neutral lipids with lower lipid toxicity for further processing. Lipid deposition induced by KLF16 knockdown might achieve lipid digestion through the upregulation of liver outward transport of lipid droplets or the promotion of mitochondrial β-oxidation after the overexpression of ATF4 [31,32]. At present, downstream lipid metabolism genes affected by ATF4 are still in the exploration stage.

This study provides a new explanation for the emergence of MAFLD and provides a new entry point for effective treatment of the disease. For MAFLD patients in the early stage, patients can be divided into groups based on the liver KLF16 expression level to predict disease progression and prognosis. Through gene therapy for MAFLD, patients can increase the expression of KLF16 in the liver to improve liver tolerance to lipid deposition.

By now, our research still has limitations. This study has not further explored the effect of KLF16 expression change on intracellular lipid composition and proportion in MAFLD hepatocytes. Further studies are needed to verify whether KLF16 has the same or opposite direct regulatory effects on other ER stress-related proteins. Phenotypic validation in human liver cells has not been performed in this study. In future studies, we will combine the lipid omics changes in the liver after KLF16 knockdown and the changes in lipid metabolism-related molecules for a combined analysis, hypothesis, and exploration.

## Conclusion

In conclusion, the results of this study suggest that the upregulation of KLF16 may act as a spontaneous protective mechanism and be involved in regulating lipid metabolism in the liver in MAFLD. The upregulation of KLF16 promotes the expression of ATF4 at the transcriptional level, maintains the activation of the PERK-EIF2α-ATF4 pathway during endoplasmic reticulum stress, promotes the expression of downstream lipid transportation-related genes, fatty acid β-oxidization-related genes, and lipid synthesis-related genes, facilitates the entry of deposited lipids into the dynamic cyclic metabolic process, and improves lipid deposition lesions. This approach highlights the potential of KLF16 as a key factor in the complex regulatory network for alleviating lipid-induced liver stress, providing a promising approach for future therapeutic interventions in MAFLD.

## Supplementary Material

Supplemental Material

Supplemental Material

FigureS2.tif

FigureS1.tif

## Data Availability

The authors declare that the data supporting the findings of this study are available on request from the corresponding author, [Wenfang Peng], upon reasonable request.
