# Supplementary material for: The role of the transcription factor KLF16 in metabolic dysfunction associated fatty liver disease: regulatory linkages between lipid deposition and the expression of ATF4
Source: Ann Med. 2025 Oct 1;57(1):2566872. doi: 10.1080/07853890.2025.2566872 (PMC12490409; doi:10.1080/07853890.2025.2566872)
Supplement: Supplemental Material [file IANN_A_2566872_SM4843.docx]

**FigureS1. The expression of KLF16 was up-regulated in NAFLD models.** (**A-B**) relative proteins expression involved in ER stress in HepG2 and primary hepatocyte from Control or OA induced subjects (n=3). Statistical analysis: Data were analyzed using an unpaired two-tailed Student’s t-test to assess statistical significance. Data are expressed as mean ± SD. *p<0.05, **p<0.01, ***p<0.001.

**FigureS2. Transcriptional factor KLF16 regulate expression of ATF4.** (**A-B**) relative proteins expression involved in ER stress in HepG2 and primary hepatocyte from OA or OA+siRNA-KLF16 induced subjects (n=3). Statistical analysis: Data were analyzed using an unpaired two-tailed Student’s t-test to assess statistical significance. Data are expressed as mean ± SD. *p<0.05, **p<0.01, ***p<0.001.

**TableS1. Primer sequence and Antibody information used in the experiment. (A**) Primer sequence information in mouse. **(B**) Primer sequence information in human. (**C**) Antibody information used in the experiment.
