# Supplementary material for: The role of the transcription factor KLF16 in metabolic dysfunction associated fatty liver disease: regulatory linkages between lipid deposition and the expression of ATF4
Source: Ann Med. 2025 Oct 1;57(1):2566872. doi: 10.1080/07853890.2025.2566872 (PMC12490409; doi:10.1080/07853890.2025.2566872)
Supplement: Supplemental Material [file IANN_A_2566872_SM4836.docx]

TableS1

A Primers sequence information used in the experiment

| **Gene(mouse)** | **Primer sequence** |
| --- | --- |
| GAPDH | CAGGAGAGTGTTTCCTCGTCC  TGAGGTCAATGAAGGGGTCG |
| β-actin | ACTGTCGAGTCGCGTCCA  ATCCATGGCGAACTGGTGG |
| KLF16 | CGCTTCGTCTCCCTCTTCG  AAGGGCCGCTCACCTGT |
| ATF4 | CGCCTTGTAAGACACCGGAA  GGTGGCCGCAAGCCTTTAT |
| FABP1 | TGAAGGCAATAGGTCTGCCC  CAGGGTGAACTCATTGCGGA |
| CD36 | GCTGATTACTTCTGTGTACTGCC  TTGAAAGGCTAGGAAACCATCCA |
| FATP2 | TCGTGGAGGTCTGAAGTCAC  CCGCTTTTGGAAGACCTGTG |
| FATP5 | TAACGTCCCTGAGCAACCAG  ACACATTTGCCCGAAGTCCA |
| ACSL4 | GAAAGCAAACTGAAGGCGGC  GCTCCCAGCTCCTCTACTGA |
| CPT1 | TCTGGCTCTATGAGGGCTCG  CTGCCCACTCTACCCTTCCT |
| MCAD | TGACAAAAGCGGGGAGTACC  CCATACGCCAACTCTTCGGT |
| LCAD | TGCACACATACAGACGGTGC  CATGGAAGCAGAACCGGAGT |
| ATGL | CCTTAGGAGGAATGCCCTGC  CTCCAGCGGCAGAGTATAGG |
| MAGL | CGGAACAAGTCGGAGGGTTC  CTGTGGAGGACGTGATAGGC |
| CPT2 | CATCGTACCCACCATGCACTA  CACAACACTTCTGTCTTCCTGA |
| APOA | AAAGACAGCGGCAGAGACTA  GTTTTCCAGGAGATTCAGGTTCAG |
| APOB | TACTTCCACCCACAGTCCCCT  TGCTTTTTAGGGAGCCTAGCA |
| APOC1 | TGAGAGATCCTTAGATCCAGGGTG  GCCTTCCAAGGTCATGGCTA |
| APOC2 | GCATGGGGTCTCGGTTCTT  GTTCCCCTGGACCTCATTTCC |
| APOC3 | GCTTGGGACTCATGGTACG  CTCTACCTCTTCAGCTCGGG |
| APOE | AAAGCAGGACTTAGCCGGGA  CTCGGCTAGGCATCCTGTCA |
| ACSS2 | TATGTGACCGGAGATGGCTG  AAGTGCCGATTCCACCTCTG |
| ACLY | GCTATGCCCCAAGGAAAGAGT  CTCGGGAACACACGTAGTCA |
| ACC1 | CCGCCAGCCTGAGTTCTTTT  ATCGGGAGTGCTGGTTTAGC |
| ACC2 | ATGTGGAGCATCCCTCAAGT  CACTACCATGTTCCTCGTCTG |
| FASN | GACTCGGCTACTGACACGAC  CGAGTTGAGCTGGGTTAGGG |
| GPAT | AGCTTCTAAGTCACCCACACC  GTCCCAAACCATGTGCTGTAT |
| DGAT | TGTGAGCCCCGACTTGG  GCAGACGATGGCACCTCA |
| SCD | GCCCCAAACGCCACAACTTTA  GTGTACGAAGGCGTCATCTCTTC |
| FAS | GTCAACCATGCCAACCTGAAAA  AATCACTCCAACGGGCTGAA |
| ELOVL6 | ACCGCAAGGCATTCATTTCC  AGTCGCTACGTGTTCTCTGC |
| SREBP1 | TCCAGCAGGTCCCAGTTGT  AGGAGCCAGGGTGCTGAT |
| CHREBP | CCTGAGCATCTGCAGCCTC  GAGACCAGCTTGCCACTGAG |
| HMGCR | ACGTGGTGTGTCTATTCGCC  CAAGCTCCCATCACCAAGGA |
| PPARα | GTGCATTTGGGCGTATCTCAC  GAACTTCAACTTGGCTCTCCTC |

B Primers sequence information used in the experiment

| **Gene(human)** | **Primer sequence** |
| --- | --- |
| GAPDH | GAAAGCCTGCCGGTGACTAA  GCCCAATACGACCAAATCAGAG |
| β-actin | ACAGAGCCTCGCCTTTGC  GATATCATCATCCATGGTGAGCTGG |
| KLF16 | GGACTGCGCCAAAGCCTA  AAGGGCGTTCCCCTGTG |
| ATF4 | AACAACAGCAAGGAGGATGC  TACCCAACAGGGCATCCAAG |
| FABP1 | ATCGTGCAGAATGGGAAGCA  TCCAACTGAACCACTGTCTTGA |
| CD36 | AGGGAAGAGAGATGAGGAACCA  TCCGGTCACAGCCCATTTTT |
| FATP2 | AGCGGATTGAAGGCAGATGA  AAGGCAAGAGTAGCACCAGC |
| FATP5 | TGTTGTCGCAGGTGGACTTC  CTTACCCTCACAACCTGGCA |
| ACSL4 | TTTTTGCGAGCTTTCCGAGTG  GCCGACAATAAAGTACGCAAATG |
| CPT1 | GACTCTGGAAACGGCCAACT  GAGCAGCACTTTCAGGGAGT |
| MCAD | TATTGTCCGAGTGGCCGGAA  AGGACCTGTAAAGAACACCTGC |
| LCAD | CCGAGTCGCCGAGTAGC  TGAGAACATCGCGCGGC |
| ATGL | TGTCCTTCACCATCCGCTTG  GTCTGCTCCTTCATCCACCG |
| MAGL | CTGGTCAATGCAGACGGACA  ACACAAAGATGAGGGCCTTGG |
| CPT2 | ACAGTGCTGGTGAGCTTCAG  TCAAAGCCCTGGCCCATTG |
| APOA | CTGAGCAACAATGCCAAGGAG  CTGGAAGAGGGCATTGAGTTG |
| APOB | ACCGGGGACACCAGATTAGA  AGGGTATCCACCAAGGCTCT |
| APOC | GTGGGGAAAGGGACTAAGGTG  GCAGGACCTTTATCAGGCTCT |
| APOE | GACCCGCTAGAAGACTGGC  GAATGTGACCAGCAACGCAG |
| ACSS2 | CCTAAAACCCGCTCAGGGAA  GTTCACTGGATGGTCAGGCA |
| ACLY | GACTTCGGCAGAGACAGGTAG  AGGAGTTCTTTGCCCGTCTG |
| ACC1 | GTTGCCACCCTGAGGTCTTT  GGCCAAGGGAGATGGTTCAT |
| ACC2 | CGAGACATCTGGAACTCGGA  CCAATAGCTCCCAAGCTGAG |
| FASN | GTCTTGAACTCCTTGGCGGA  AGGAAGATAGCCATGCCGAG |
| GPAT | CTGAACTCTGAGATGCTGCG  TCCTGAGACTCTGACGCTCT |
| DGAT | AGAGGAGGTGCGGGAC  GCGATGGCACCTCAGC |
| SCD | CCCGACGTGGCTTTTTCTTC  GCCAGGTTTGTAGTACCTCCTC |
| FAS | TTTTCTTGGGCCTTGATGCG  GCAACAGACGTAAGAACCTCAA |
| ELOVL6 | GCACCCGAACTAGGAGATACA  ACAGGAGCACAGTGATGTGG |

| **Name** | **Brand** | **species** |
| --- | --- | --- |
| KLF16 monoclonal antibody | Santa cruz | mouse |
| ATF4 monoclonal antibody | CST | rabbit |
| PERK monoclonal antibody | CST | rabbit |
| p-PERK monoclonal antibody | CST | rabbit |
| IRE1-α monoclonal antibody | CST | mouse |
| ATF6 monoclonal antibody | CST | rabbit |
| CHOP monoclonal antibody | CST | mouse |
| GAPGH monoclonal antibody | abcam | rabbit |
| β-actin monoclonal antibody | abcam | rabbit |
| β-tublin monoclonal antibody | Santa Cruz | mouse |
| XBP1 monoclonal antibody | CST | rabbit |
| eIF2α monoclonal antibody | CST | rabbit |
| BIP monoclonal antibody | CST | mouse |
| Anti-rabbit secondary antibody | CST |  |
| Anti-mouse secondary antibody | CST |  |

C Antibody information used in the experiment
