# Supplementary figures and images for: The role of the transcription factor KLF16 in metabolic dysfunction associated fatty liver disease: regulatory linkages between lipid deposition and the expression of ATF4
Source: Ann Med. 2025 Oct 1;57(1):2566872. doi: 10.1080/07853890.2025.2566872 (PMC12490409; doi:10.1080/07853890.2025.2566872)

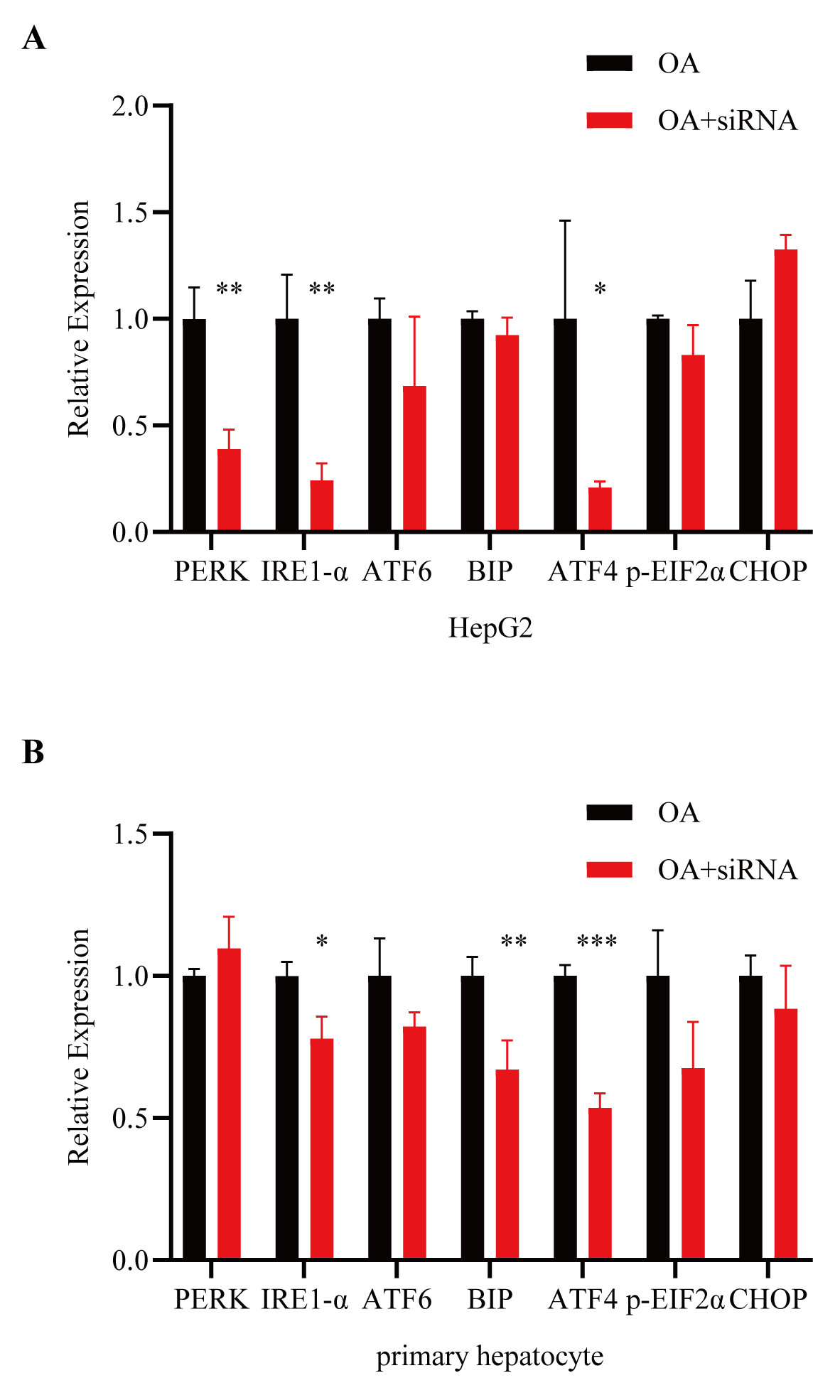

Supplement: FigureS2.tif [file IANN_A_2566872_SM3673.tif]

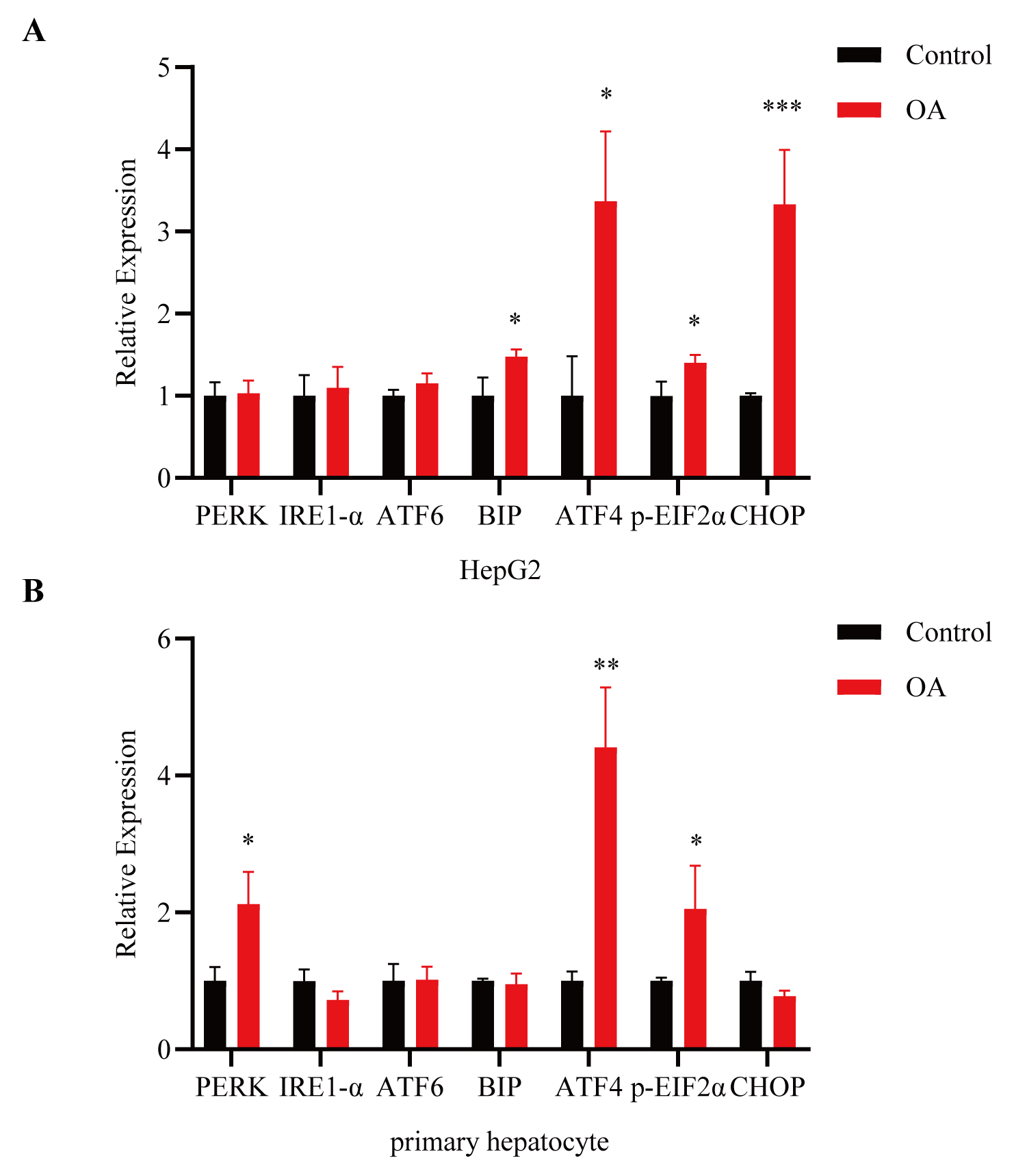

Supplement: FigureS1.tif [file IANN_A_2566872_SM3672.tif]
